# Pathophysiological Bases of Comorbidity in Migraine

**DOI:** 10.3389/fnhum.2021.640574

**Published:** 2021-04-20

**Authors:** Claudia Altamura, Ilenia Corbelli, Marina de Tommaso, Cherubino Di Lorenzo, Giorgio Di Lorenzo, Antonio Di Renzo, Massimo Filippi, Tommaso B. Jannini, Roberta Messina, Pasquale Parisi, Vincenzo Parisi, Francesco Pierelli, Innocenzo Rainero, Umberto Raucci, Elisa Rubino, Paola Sarchielli, Linxin Li, Fabrizio Vernieri, Catello Vollono, Gianluca Coppola

**Affiliations:** ^1^Headache and Neurosonology Unit, Neurology, Campus Bio-Medico University Hospital, Rome, Italy; ^2^Clinica Neurologica, Dipartimento di Medicina, Ospedale S.M. Misericordia, Università degli Studi di Perugia, Perugia, Italy; ^3^Applied Neurophysiology and Pain Unit, SMBNOS Department, Bari Aldo Moro University, Policlinico General Hospital, Bari, Italy; ^4^Department of Medico-Surgical Sciences and Biotechnologies, Sapienza University of Rome Polo Pontino, Latina, Italy; ^5^Laboratory of Psychophysiology and Cognitive Neuroscience, Department of Systems Medicine, University of Rome Tor Vergata, Rome, Italy; ^6^IRCCS–Fondazione Santa Lucia, Rome, Italy; ^7^IRCCS–Fondazione Bietti, Rome, Italy; ^8^Neuroimaging Research Unit, Division of Neuroscience, Institute of Experimental Neurology, Milan, Italy; ^9^Neurology Unit, IRCCS San Raffaele Scientific Institute, Milan, Italy; ^10^Vita-Salute San Raffaele University, Milan, Italy; ^11^Child Neurology, Department of Neuroscience, Mental Health and Sense Organs (NESMOS), Faculty of Medicine & Psychology, c/o Sant'Andrea Hospital, Sapienza University, Rome, Italy; ^12^Headache Clinic, IRCCS–Neuromed, Pozzilli, Italy; ^13^Neurology I, Department of Neuroscience “Rita Levi Montalcini,” University of Torino, Torino, Italy; ^14^Department of Emergency, Acceptance and General Pediatrics, Bambino Gesù Children's Hospital, Scientific Institute for Research, Hospitalization and Healthcare (IRCCS), Rome, Italy; ^15^Nuffield Department of Clinical Neurosciences, Centre for Prevention of Stroke and Dementia, John Radcliffe Hospital, University of Oxford, Oxford, United Kingdom; ^16^Department of Neurology, Fondazione Policlinico Universitario “Agostino Gemelli” IRCCS, Catholic University, Rome, Italy

**Keywords:** CNS disorders, thalamocortical network dysexcitability, trigeminovascular system, migraine threshold, energetic balance

## Abstract

Despite that it is commonly accepted that migraine is a disorder of the nervous system with a prominent genetic basis, it is comorbid with a plethora of medical conditions. Several studies have found bidirectional comorbidity between migraine and different disorders including neurological, psychiatric, cardio- and cerebrovascular, gastrointestinal, metaboloendocrine, and immunological conditions. Each of these has its own genetic load and shares some common characteristics with migraine. The bidirectional mechanisms that are likely to underlie this extensive comorbidity between migraine and other diseases are manifold. Comorbid pathologies can induce and promote thalamocortical network dysexcitability, multi-organ transient or persistent pro-inflammatory state, and disproportionate energetic needs in a variable combination, which in turn may be causative mechanisms of the activation of an ample defensive system with includes the trigeminovascular system in conjunction with the neuroendocrine hypothalamic system. This strategy is designed to maintain brain homeostasis by regulating homeostatic needs, such as normal subcortico-cortical excitability, energy balance, osmoregulation, and emotional response. In this light, the treatment of migraine should always involves a multidisciplinary approach, aimed at identifying and, if necessary, eliminating possible risk and comorbidity factors.

## Introduction

It is commonly accepted that migraine is a pathology of the nervous system. For many years, attention has been focused on the predominant role of the brainstem in the genesis of migraine attacks and, probably, in its recurrence (Weiller et al., 1995; Bahra et al., 2001; Stankewitz et al., 2011). This role of the brainstem is closely linked to its physiological actions such as its ability to set the signal-to-noise ratio of cortical activity directly or indirectly through the thalamus (Mesulam, 1990), to control the neuro-vascular coupling at the cortical level (Raichle et al., 1975; Goadsby et al., 1982; Edvinsson et al., 1983), probably playing a role in the unleashing of the migraine aura, and its contribution in the development of the central sensitization processes (Zambreanu et al., 2005; Lee et al., 2008). The latter action is likely to be mediated both by the caudal trigeminal nucleus and by other brainstem nuclei and is at the basis of some clinical manifestations of episodic migraine (EM) and chronic migraine (CM), such as phono/photo-phobia and osmophobia (Okamoto et al., 2009; Stankewitz et al., 2011; Joffily et al., 2016). More recently, however, functional neuroimaging studies renewed the interest in the hypothalamus as the possible generator of migraine. They showed that the hypothalamus activates shortly before the beginning of migraine attack, during the period in which some patients experience premonitory symptoms, and during the attack, it displays altered connection with the spinal trigeminal nucleus (Schulte and May, 2016; Schulte et al., 2020). The hypothalamus and the brainstem are not the only brain structures involved in the pathophysiology of migraine. There is various evidence of functional and structural abnormalities of the thalamus and thalamus–cortical fiber bundles in migraineurs, especially between attacks when the patient has no pain but is in the potency of its recurrence (Coppola et al., 2005, 2014; DaSilva et al., 2007; Rocca et al., 2008). As for the brainstem, also the thalamus may contribute to the clinical manifestation of migraine (Burstein et al., 2010; Noseda et al., 2010; Russo et al., 2014). Both functional and structural abnormalities have been consistently detected also at the cortical level, predominantly in the visual areas (Puledda et al., 2019), but no cortex has been spared, not even the cerebellar one (Coppola et al., 2020).

The peripheral nervous system is also evidently involved. This is the case of the sensory afferences of the first branch of the trigeminal nerve that innervate the small meningeal arteries to form the trigeminal–vascular system. Various scientific evidence suggests that the migraine attack begins at that level, i.e., with the release of the vasoactive polypeptide calcitonin gene-related peptide (CGRP) and the consequent triggering of the so-called peripheral sensitization (Burstein et al., 2000a). The latter consists in the release at a peripheral level of pro-inflammatory substances that sensitize the meningeal nociceptors and constitute a neurogenic pro-inflammatory state, which, if it persists long enough, triggers the aforementioned central sensitization and, therefore, the procession of symptoms and neurological signs that accompany migraine pain (Edvinsson, 2019). The animal model shows that the activation of first-order neurons of the trigeminal–vascular system can be evoked by cortical spreading depression (CSD), an electrocortical phenomenon thought to be at the base of the migraine aura (Bolay et al., 2002).

This important and widespread involvement of the central and peripheral nervous system is sustained by genetics. Unfortunately, the genetics of rare, familiar forms of migraine with hemiplegic aura does not seem to be the same as the most common forms of migraine with and without aura (Hovatta et al., 1994; Monari et al., 1997; Kim et al., 1998; Jones et al., 2001; Brugnoni et al., 2002; Noble-Topham et al., 2002; Wieser et al., 2003). But more recent genome-wide association studies carried out on a large cohort of migraine patients have identified a number of loci associated with the risk of migraine. These loci show enrichment for genes expressed in vascular and muscular tissues (Gormley et al., 2016), as well as for genes involved in glutamate homeostasis, synaptic plasticity, and pain-related pathway (Chasman et al., 2011), However, metabolic aspects should not be overlooked. In fact, evidence from neuroimaging (Sándor P. et al., 2005; Lodi et al., 2006; Lisicki et al., 2018) and genetic (Sparaco et al., 2006; Di Lorenzo et al., 2009) studies, as well as controlled pharmacological trials (Schoenen et al., 1998; Sândor P. S. et al., 2005), shows how mitochondrial energy metabolism can be altered in migraine and can predispose to the recurrence of attacks.

The simultaneous presence of multiple comorbidities can further complicate the clinical and prognostic presentation of migraine. Various disorders can occur as comorbidities with migraine and include neurological, psychiatric, cardio- and cerebrovascular, gastrointestinal, metaboloendocrine, painful, and immunological conditions. Each of these has its own genetic load and shares some common characteristics with migraine. In fact, all the aforementioned pathologies are associated with migraine in both adults and children (Scher et al., 2003b, 2005, 2008; El-Metwally et al., 2004; Buse et al., 2019). For these reasons, some researchers believe that there may be a common genetic background that predisposes some people to migraines and other comorbidities (Burch et al., 2019).

In this article, we review, narratively, published data describing these migraine comorbidities, and then we further discuss available evidence for their shared pathophysiological mechanisms.

## Cerebrovascular Dysfunction and Migraine

### Scientific Evidence of Comorbidity and Pathophysiological Links

A meta-analysis including over a million subjects concluded that migraineurs present an increased long-term risk of cardiovascular and cerebrovascular events (Mahmoud et al., 2018). The relative risk varies from 1.56 to 2.41 in migraine with aura (MA) to 1.11–1.83 in migraine without aura (MO) (Øie et al., 2020).

The physiopathological link between stroke and migraine is multifaceted: different aspects from thromboembolism, hemodynamic dysfunction, to energetic failure. They each act as part of a puzzle piece.

#### Thrombosis and Embolism

Clinical atherosclerosis has been cleared as being responsible for the increased vascular risk in migraine patients, but some studies reported that subclinical atherosclerosis (i.e., intima-media thickening) could be a marker of endothelial dysfunction, linking vascular disease to migraine (Stam et al., 2013; Van Os et al., 2017; Magalhães and Sampaio Rocha-Filho, 2018; Magalhães et al., 2019; Yilmaz Avci et al., 2019). Nitric oxide (NO), endothelin-1, von Willebrand factor, plasminogen activator inhibitor-1, angiotensin II, prostacyclin, and platelet-activating factor are among the substances secreted by the endothelium in reaction to local environment changes, which can result in local inflammation and thrombosis. This phenomenon is defined as endothelial activation (Boulanger, 2018).

The endothelial activation was found guilty of predisposing patients with migraine to vascular diseases.

A pro-inflammatory and pro-coagulative milieu was consistently demonstrated in migraineurs, particularly in MA, CM, and women, predominantly in the premenopausal period (Liman et al., 2015; Ferroni et al., 2017; Tietjen and Collins, 2018). Nevertheless, genetic studies on polymorphism for thrombophilic mutations were not consistent; although some reported an increased prevalence of pro-thrombotic polymorphisms (Lippi et al., 2015; Cecchi et al., 2018), a definitive conclusion is difficult to draw (Malik et al., 2016). High estrogen state is probably the most significant factor associated with stroke occurrence in migraine, especially if accompanied by cigarette smoking, particularly in MA patients (Kurth et al., 2012). Finally, platelet activation has been suggested as another possible intermediary to explain the increased vascular risk via augmented aggregation and interaction with leucocytes (Borgdorff and Tangelder, 2012; Danese et al., 2014). Supporting this evidence, antiplatelet therapy seems to relieve MA (Turk et al., 2017), also in patients without patent foramen ovale (PFO) (Altamura et al., 2019b).

It is not completely understood if migraine attacks determine endothelial activation as the results of neural activation and oxidative stress or the other way around: transient hypoperfusion due to the pro-inflammatory and pro-thrombotic states may favor neural distress, inducing migraine (Dalkara et al., 2010). In this scenario, the high prevalence of PFO observed in MA patients offers the pathway through which micro-emboli can reach the cerebral circulation (Del Sette et al., 1998). Interestingly, in the Oxford Vascular Study cohort, migraine was the factor most strongly associated with cryptogenic TIA and ischemic stroke, suggesting a causative role for migraine or a shared etiopathogenesis (PFO?) (Li et al., 2015). As a further complication, migraine with visual aura is a risk factor for atrial fibrillation (Sen et al., 2018), while the relation between migraine and carotid artery dissection is still elusive, although they may share a common genetic substrate (Malik et al., 2016; De Giuli et al., 2017; Kok et al., 2018).

#### Hemodynamic Dysfunction

In addition to monogenic diseases with cerebral arteriopathy and migraine typical features [i.e., cerebral autosomal dominant arteriopathy with subcortical infarcts and leukoencephalopathy (CADASIL)], a meta-analysis of susceptibility genes for migraine identified enrichment for genes expressed in vascular and smooth muscle tissues, consistent with a vascular involvement (Gormley et al., 2016).

Cerebral hemodynamics is a complex system that allows adequate brain perfusion also in conditions that pose cerebral blood supply at risk. It relies on the orchestral action of neurogenic, myogenic, endothelial, and metabolic responses.

The neurogenic control is achieved by neurotransmitters with vasoactive properties [CGRP, serotonin, pituitary adenylate cyclase-activating polypeptide (PACAP), and NO] released by sympathetic, parasympathetic, and sensory neurons and in smaller arterioles in response to the neuronal firing (Frederiksen et al., 2019). These neurotransmitters have a key role also in migraine attacks. Similarly, the endothelium plays a significant role in vessel caliper regulation via the paracrine secretion of substances such as NO, adrenomedullin, and endothelin-1 that have been largely involved in migraine physiopathology (Kis et al., 2003; Tietjen and Collins, 2018). The myogenic control regulates vessel caliper in response to change in transmural pressure (i.e., autoregulation), while the metabolic response allows vasodilation following the local increase in H+ concentration [vasomotor reactivity (VMR)].

Cerebral VMR is a marker of hemodynamic efficiency and correlates with stroke risk (Reinhard et al., 2014). During migraine attacks, particularly in the aura phase of MA, VMR, and neurovascular coupling are impaired as demonstrated by experimental studies on CSD (Harer and von Kummer, 1991; Ayata and Lauritzen, 2015). Conversely, in the interictal period, most studies reported a preserved or higher VMR in migraineurs compared with controls, especially in MA patients (Thomsen et al., 1995; Silvestrini et al., 1996, 2004; Valikovics et al., 1996; Kastrup et al., 1998; Fiermonte et al., 1999; Dora and Balkan, 2002; Vernieri et al., 2008; Chan et al., 2009; Altamura et al., 2019a, 2020), with some exceptions suggesting an impaired VMR mainly in the posterior circulation (Totaro et al., 1997; Silvestrini et al., 2004; Perko et al., 2011b; Rajan et al., 2014). To note, cerebral VMR seems to be less effective in CM (Akgün et al., 2015; González-Quintanilla et al., 2015). Moreover, estrogen use was associated with lower VMR in MA patients, curtailing their hemodynamic resources (Altamura et al., 2019b).

Cerebral autoregulation was investigated by obtaining controversial results for the anterior circulation (Müller and Marziniak, 2005; Reinhard et al., 2007), while it resulted in impairment in the posterior circulation only in MA patients (Reinhard et al., 2012).

How and whether the endothelium activation is implicated in this abnormal hemodynamics are a matter of several investigations (Yetkin et al., 2006; Vanmolkot et al., 2007; Napoli et al., 2009; Butt et al., 2015; Heshmat-Ghahdarijani et al., 2015). The endothelial reactivity can be studied peripherally by brachial artery flow-mediated dilation (FMD), which reflects the arterial tone self-regulation mediated by the endothelium in response to changes in the local environment (Tremblay and Pyke, 2018). An altered FMD is associated with a higher vascular risk (Shechter et al., 2014). Most studies suggest that FMD is preserved or increased in episodic MO and MA (Vanmolkot and de Hoon, 2010; Vernieri et al., 2010; Perko et al., 2011a; Larsen et al., 2016; Altamura et al., 2018, 2020) and reduced in CM (González-Quintanilla et al., 2015).

In summary, cerebral hemodynamics in the anterior circulation is preserved or hyper-reactive in migraine patients and especially in MA, supporting mostly a protective rather than impaired hemodynamics. Moreover, the hemodynamic efficiency seems to improve over time in MA patients (Gollion et al., 2019), possibly as the result of frequent ischemic threats (i.e., ischemic preconditioning). On the other hand, VMR may be impaired during attacks, and both cerebral and peripheral hemodynamics seem to be altered in the chronic condition. Finally, the frequent use of triptans or ergots can disrupt the hemodynamic balance toward vasoconstriction (Roberto et al., 2014).

#### Energetic Failure

The migraine brain seems to be easy prey for vascular insults. Phylogenetically, CSD can be interpreted as a metabolic reset of cerebral activity occurring when energetic demands overcome the resources, aiming at restoring homeostasis and reducing harmful oxidative stress levels (Meldrum Robertson et al., 2020). However, the criticality is not the scarce energy supply but its excessive requirement, due to the transient or persistent sensory hypersensitivity and its inefficient use. Several evidence supports this hypothesis: from the genetic link where mitochondrial disturbances and migraine coexist [e.g., mitochondrial encephalopathy, lactic acidosis, and stroke-like episodes (MELAS)] to the common observation that being starved is an important trigger for migraine attacks. The energetic frailty of the migraine brain makes it particularly vulnerable to ischemic damage. Familial hemiplegic migraine (FHM)1 transgenic mouse models present a more rapid expansion of infarct volumes and larger perfusion deficits (Eikermann-Haerter et al., 2012). The same observation was clinically made in humans: among stroke patients, migraineurs, and in particular those with MA, displaying a reduced ratio between infarcted and hypo-perfused tissue (Mawet et al., 2015; Pezzini et al., 2018). Besides, patients with migraine present more often with cortical infarcts (Øygarden et al., 2014).

These findings strongly support the susceptibility of the migraine brain (mainly with aura) to milder ischemic conditions.

In summary, when investigating the case of the migraine–stroke connection, we should look for a criminal conspiracy where all the suspects of the neuro-vascular-endothelial unit have a guilty role.

## Metabolic and Endocrine Comorbidities of Migraine

Metabolic diseases, like diabetes and obesity, as well as endocrine diseases are highly prevalent conditions in the general population. Recently, several studies showed the presence of a complex and intriguing comorbidity between migraine and these disorders, suggesting new pathogenetic mechanisms for migraine.

### Scientific Evidence of Comorbidity

#### Insulin Resistance, Metabolic Syndrome, and Migraine

Insulin resistance (IR) is a condition characterized by a subnormal physiological response to normal insulin concentrations, with increased quantities of insulin produced to maintain adequate intracellular glucose concentrations. Metabolic syndrome (MetS) is a syndrome characterized by a cluster of metabolic abnormalities including hyperglycemia, hypertension, dyslipidemia, abdominal obesity, and a pro-inflammatory state. These two medical conditions are interrelated and share common underlying mediators and pathways.

Since the first description in 2005 (Rainero et al., 2005), several studies showed the presence of an association among migraine, IR, and MetS (Cavestro et al., 2007; Bhoi et al., 2012; Fava et al., 2014). Glucose plasma concentrations are significantly increased during spontaneous migraine attacks (McCarthy et al., 2001). Hyperinsulinemia is associated with a 5.7-fold higher risk for migraine (Netzer et al., 2008). Patients with CM are more insulin resistant than patients with EM and controls (Zhang et al., 2020). In comparison with healthy controls, patients with MA are at higher risk of MetS [odds ratio (OR) = 3.45; 95% CI: 1.63–7.29], while MO individuals are not (Gruber et al., 2010). A recent study showed that MetS is significantly associated with CM (OR = 5.342, *p* = 0.032), and the risk for MetS increases significantly in patients with CM and medication-overuse headache (OR = 12.68, *p* = 0.007; Fava et al., 2014). Furthermore, genetic studies provided evidence that polymorphisms of the insulin-receptor gene (INSR) are associated with migraine (He et al., 2015; Streel et al., 2017). A recent systematic review of the observational studies linking the MetS with migraine has identified several weaknesses in the available research and suggested the need for future investigations using more rigorous methodology (Andreeva et al., 2019). However, a modulation of the metabolic pathway linked to insulin metabolism might be of relevance in migraine prophylaxis.

#### Diabetes and Migraine

Several studies investigated the relationship between diabetes and migraine. Epidemiological data showed that migraine patients are not at increased risk of developing and type 2 diabetes mellitus (T2DM) (López-De-Andrés et al., 2018). Data from the Nord-Trøndelag Health Surveys showed that patients with type 1 diabetes showed a lower prevalence of migraine (OR = 0.47, 95% CI: 0.26–0.96) than did subjects without DM (Hagen et al., 2018).

A recent study showed a lower risk of T2DM in women with active migraine compared with women with no migraine history [univariate hazard ratio, 0.80 (95% CI: 0.67–0.96)]. Furthermore, the authors found a linear decrease in the prevalence of active migraine during the 24 years before a diagnosis of T2DM (Fagherazzi et al., 2019).

Biological mechanisms underlying the protective effect of diabetes on the risk of developing migraine attacks are, at present, unclear.

#### Obesity and Migraine

Overweight and obesity are both highly prevalent medical conditions, associated with substantial personal and societal impact. Population studies have consistently identified an association between obesity, headache, and particularly migraine (Peterlin et al., 2010; Pavlovic et al., 2017; Kristoffersen et al., 2020). A recent meta-analysis, encompassing 288,981 participants in 12 different studies, showed that the age- and sex-adjusted pooled risk for migraine in obese patients is increased by 27% in comparison with those of normal weight (OR = 1.27; 95% CI: 1.16–1.37, *p* < 0.001). In underweight individuals, the pooled risk of migraine was marginally increased by 13% (OR = 1.13; 95% CI: 1.02–1.24, *p* < 0.001; Gelaye et al., 2017). Plasma concentrations of adipokines, like leptin, adiponectin, and resistin, are significantly increased in both EM and CM, suggesting a role for these pro-inflammatory mediators in the comorbidity between obesity and migraine (Peterlin et al., 2016; Rubino et al., 2017).

Comorbidity between migraine and obesity as well as the role of several dietary factors in headache attacks prompted the investigations of different dietary regimens for migraine prevention (Gazerani, 2020; Hindiyeh et al., 2020). Finally, the ketogenic diet, a diet that leads to the elevation of ketone bodies, has shown great promise in the prevention of migraines (Di Lorenzo et al., 2015, 2019b).

#### Hypothyroidism and Migraine

Hypothyroidism is a frequent medical condition with a lifetime prevalence of 2%. Several studies showed an association between migraine and hypothyroidism, in both adolescents and adults, with hypothyroidism being significantly more prevalent in subjects with CM compared with those with EM (Fallah et al., 2012; Spanou et al., 2019). Data from the Fernald Medical Monitoring Program in the USA demonstrated that headache disorders are risk factors for the development of new-onset hypothyroidism, with migraine patients showing an increased risk of 41% of developing this disorder (Martin et al., 2017). A recent, case–control study showed that patients with subclinical hypothyroidism have an increased risk of developing migraine (Rubino et al., 2019). Taken together, these studies suggest that migraine and hypothyroidism are linked by a bidirectional relationship. Genetic and immune mechanisms may explain this association.

#### Endometriosis and Migraine

Observational studies indicated that migraine and endometriosis co-occur within individuals more than expected by chance (Yang et al., 2012). A recent systematic review found a significant association between endometriosis and the risk of migraine (OR = 1.56; 95% CI: 1.21–1.90) (Jenabi and Khazaei, 2020). The analysis of endometriosis phenotypes showed that ovarian endometrioma and deeply infiltrating endometriosis were significantly more frequent in migraine female patients than in controls (OR = 2.78; 95% CI: 1.11–6.98 and OR = 2.51; 95% CI: 1.25–5.07, respectively) (Maitrot-Mantelet et al., 2020). The biological mechanisms underlying this comorbidity remain unknown. Interestingly, a recent genome-wide association study found a positive and highly significant genetic correlation (*p* = 2.30 × 10^−25^) between endometriosis and migraine and suggested a role for genes involved in interleukin-1 receptor binding, focal adhesion-PI3K-Akt-mTOR-signaling, mitogen-activated protein kinase (MAPK), and tumor necrosis factor-alpha (TNF-α) signaling in the association between these two traits (Adewuyi et al., 2020).

### Supposed Pathophysiological Mechanisms

The pathophysiological mechanisms underlying the complex association between migraine, metabolic, and endocrine disease are still under investigation; and additional, rigorous studies are needed. However, some suggestions are of particular interest.

Investigations of metaboloendocrine comorbidities of migraine further support the role of shared molecular genetic mechanisms between all these highly prevalent medical conditions. Migraine and diabetes, obesity, and endometriosis are complex genetic traits sharing common genes as well as common metabolic pathways (McCarthy et al., 2001; Netzer et al., 2008; Adewuyi et al., 2020). Further investigating these pathways will allow us to disentangle the biochemical mechanisms of migraine.

An increasing amount of evidence suggests that migraine patients have a reduced cerebral energy reserve, facilitating the onset of headache attacks under stress. The study investigating the level of metabolism with fluorodeoxyglucose (FDG)-PET and the level of functional cortical activity with evoked potentials showed a low metabolism of the cortical areas and high functional activity in migraineurs compared with healthy subjects (Lisicki et al., 2018). This abnormal functional activity in migraineurs, defined as cortical hyperresponsivity (Coppola et al., 2007), can be normalized through non-pharmacological therapies as a ketogenic dietary regimen (Di Lorenzo et al., 2016, 2019a). This is consistent with the ability of ketogenic feeding of potentiating mitochondrial energy metabolism (Bough et al., 2006; Maalouf et al., 2007).

Migraineurs have altered mitochondrial functioning (Sparaco et al., 2006; Di Lorenzo et al., 2009), and drugs like riboflavin and Co-Enzyme Q10, both physiologically implicated in the mitochondrial respiratory chain, are efficacious in disease prophylaxis (Schoenen et al., 1998; Sândor P. S. et al., 2005). In this context, the study of the metaboloendocrine comorbidity of migraine supports the notion that a reduction of cerebral metabolism is a key factor in the disease pathogenesis (Lisicki and Schoenen, 2020). Binding of insulin to its receptor induces structural changes leading to auto-phosphorylation of various tyrosine residues. The final effect of insulin receptor stimulation includes translocation of the glucose transporter proteins (GLUT1 and GLUT4), promoting glucose influx in different cells. Besides, insulin receptors regulate several complex physiological actions like the synthesis and storage of carbohydrates, lipids, and protein. Dysfunction in the insulin signaling pathway may, therefore, explain the reduced metabolism observed in patients with migraine.

Insulin sensitivity is clearly impaired in migraine, even in young, non-obese, non-diabetic, normotensive patients. Plasma glucose and insulin levels increase during spontaneous migraine attacks, leading to impairment in complex metabolic patterns. Furthermore, studies with FDG-PET in migraine patients showed glucose hypometabolism in several brain areas, like the occipital, orbitofrontal, and rostral anterior cingulate cortices (Magis et al., 2017; Lisicki et al., 2018). However, the precise mechanisms of glucose metabolism impairment in migraine need to be further elucidated.

## Epilepsy and Migraine

### Scientific Evidence of Comorbidity

Epilepsy and migraine may mimic each other, and occipital lobe seizures may be easily misinterpreted as migraine with visual aura (Panayiotopoulos, 1999). The frequency of epilepsy among people with migraine (range 1–17%) is higher than in the general population (0.5–1%), just as the prevalence of migraine among patients with epilepsy is also higher than that reported in individuals without epilepsy (Lipton et al., 1994).

A cross-sectional study (Gameleira et al., 2013) conducted in adults with epilepsy showed a greater tendency of comorbidity with headaches (OR = 1.6, *p* = 0.077), which occurred in 66.1% of the cases; the highest occurrence was of migraine (32.9% of the patients), followed by tension-type headaches (TTHs) (9.2%). Yet other studies have reported a significant association with headaches, particularly migraine-type headaches, being linked to the frequency of seizures (Wang et al., 2014a,b; Mainieri et al., 2015; Mameniškienė et al., 2016; Çilliler et al., 2017). In a more recent adult large sample (15,133 subjects), migraine was also confirmed to be associated more likely with epilepsy (Buse et al., 2020). As further evidence in favor of a non-random association, a more recent systematic review (Duko et al., 2020), conducted on 5,564 study participants, reported a higher prevalence (48.4%, ranging from 46 to 52.2%) of headache among epileptic patients.

Yamane et al. (2004) found that among epileptic children, 46% of patients suffer also from headaches, of which 43.5% are classified as migraine type. In some specific childhood epilepsy syndromes (such as benign occipital epilepsy of childhood with occipital paroxysms and benign rolandic epilepsy), migraine/headaches appear to be more prevalent (Andermann and Zifkin, 1998; Clarke et al., 2009). Piccinelli et al. (2006) found electroencephalography (EEG) interictal abnormalities in 16 (12.8%) out of 137 children and adolescents with headache, particularly in those with MA. In a large, consecutive, pediatric headache population, Toldo et al. (2010) found a strong association with epilepsy; this significant strong correlation was confirmed in children (Baca et al., 2011) and adolescents (Lateef et al., 2012).

The literature shows somewhat conflicting data regarding the epidemiological aspects in the various age groups (Lipton et al., 1994; Tonini et al., 2012). This may be attributed to the co-occurrence (synergistic and/or divergent) of confounding variables adopted in the different sampling methods and study designs. These conflicting results may partly be explained by differences in the target populations, study design, age range, and methods, by inclusion criteria that are limited to referral patients with epilepsy or tertiary headache centers, by the lack of appropriate control groups, and/or by different or ill-defined diagnostic criteria (Belcastro et al., 2012; Tonini et al., 2012).

Children are more likely to have an autonomic symptomatology in both epilepsy and headache attacks (Kasteleijn-Nolst Trenité and Parisi, 2012; Parisi et al., 2012a). Moreover, they may have isolated, long-lasting ictal autonomic manifestations, while ictal autonomic manifestations (in both epilepsy and headache) in adults are usually associated, whether simultaneously or sequentially, with other motor or sensory ictal signs and symptoms (Kasteleijn-Nolst Trenité and Parisi, 2012).

So despite the limited number of studies (Yamane et al., 2004; Piccinelli et al., 2006; Toldo et al., 2010), the framework assumes markedly different shapes in the pediatric population, as stressed above, and this is, probably, why the ictal epileptic headache (IEH) (Parisi et al., 2012b) is a phenomenon that occurs with much higher probability among the infantile epileptic population (Belcastro et al., 2012).

### Supposed Pathophysiological Mechanisms

Glutamatergic (Jen et al., 2005), serotoninergic (Johnson and Griffiths, 2005), dopaminergic metabolism (Chen, 2006), and ion channel (sodium, potassium, and chloride) function might be impaired in both epilepsy and migraine (Pietrobon, 2010). In particular, it is likely that voltage-gated ion channels play a critical role in the pathways associated with migraine and epilepsy (Di Stefano et al., 2020). After blockade of either the P-/Q-type Ca^2+^ channels or the NMDA receptors, CSD cannot be induced in wild-type mouse cortical slices. By contrast, the blockade of N- or R-type Ca^2^ channels only has a slight inhibitory effect on the CSD threshold and velocity of propagation. These findings support a model according to which the initiation and propagation of the CSD involved in migraine require the influx of Ca^2+^ through pre-synaptic P-/Q-type Ca^2+^ channels, which in turn releases glutamate from the recurrent cortical pyramidal cell synapses and activates NMDA receptors (Hamberger and van Gelder, 1993; Lönnqvist et al., 2009).

CSD may be considered one of the links between headache and epilepsy (Parisi, 2009; Parisi et al., 2012a, 2013, 2015) and is characterized by a slowly propagating wave of sustained strong neuronal depolarization that generates transient intense spike activity, followed by neural suppression, which may last for minutes. As mentioned before, in animal models, CSD seems to be able to activate the trigeminovascular system, inducing the cascade release of numerous inflammatory molecules and neurotransmitters, which in humans may result in the ignition of a migraine attack (Parisi, 2009; Belcastro et al., 2011; Parisi et al., 2012a).

Both basic and clinical neurosciences support that CSD and an epileptic focus may facilitate each other, though with different extents. The required threshold is suggested to be lower for CSD than for a seizure, which would explain why it is far more likely that an epileptic patient presents a peri-ictal headache than vice versa (Parisi et al., 2008, 2012a, 2015, 2019; Parisi, 2009; Belcastro et al., 2011). The triggering causes, which may be environmental or individual (whether genetically determined or not), result in a flow of ions that mediate CSD through neuronal and glial cytoplasmic bridges rather than through interstitial spaces, as instead usually occurs in the spreading of epileptic seizures (Parisi et al., 2008, 2012a; Parisi, 2009; Belcastro et al., 2011).

Migraine and epilepsy have an important genetic component, with strong evidence pointing to a shared genetic basis between headache and epilepsy emerging from clinical/EEG and genetic studies on FHM (Di Stefano et al., 2020). Genetic variants in the same gene may be associated with migraine in some cases and with epilepsy in others. Accordingly, the genetic role as link to explain the comorbidity between headache and epilepsy and other paroxysmal disorders have also been recently underlined (Crompton and Berkovic, 2009; Ebrahimi-Fakhari et al., 2015; Galizia et al., 2015; Barbieri et al., 2019; Di Stefano et al., 2020).

Lastly, it is intriguing to stress that IEH cannot just be classified with a unique mode of pain transmission because different afferent/efferent nociceptive types of receptors and central (afferent and efferent) pathways are involved. Moreover, given the complexity of the networks involved, it is likely that the cortical projections of headache pain are widespread, also involving the areas belonging to neurolimbic network (insula, cingulate cortex, pre-frontal cortex, amygdala, and other parts of the limbic system) and not just the primary sensory-sensitive areas. This is the reason why we could consider most cases of IEH as autonomic seizures (Belcastro et al., 2011; Parisi et al., 2012a, 2019) and not just “a rare form of painful seizure,” as conversely suggested, recently, by others (Hwang et al., 2019).

## Psychiatric Comorbidity

### Scientific Evidence of Comorbidity

Migraine condition, especially when chronic, represents a huge burden, as it affects different aspects of daily living, ranging from occupation and academic to familial and social scenarios (Leonardi et al., 2005). Patients suffering from migraine might experience a higher prevalence of psychiatric comorbidities than do non-migraineurs (Burch et al., 2019). Indeed, a large body of literature shows that psychiatric disorders are highly associated with migraine, e.g., major depressive disorder (MDD), bipolar disorder (BD), post-traumatic stress disorder (PTSD), and anxiety disorders. Moreover, such comorbidities increase with the frequency of migraine episodes. Indeed, people with a 14-day or more occurrence of migraine have an adjusted OR of 6.4 for depression and 6.9 for anxiety disorders (Zwart et al., 2003). Being affected by psychiatric disorders is considered an independent modifiable factor of progression toward chronification of migraine and a tendency to medication overuse (Scher et al., 2008; Sances et al., 2010). Nonetheless, emotional distress is commonly recognized as migraine trigger (Kelman, 2007).

#### Depressive Disorders and Migraine

Depression is up to 2.5 times more prevalent in patients with migraine than in the general population, with 40% of them reporting depressive episodes during their lifetime (Lipton et al., 2000; Jette et al., 2008). As these two conditions are often comorbid, they can both lead to a higher degree of social life, family life, and career disability (Rossi et al., 2005; Bigal and Lipton, 2009). Indeed, evidence shows a consistent amount of underlying pathophysiological mechanisms shared by both disorders (Amoozegar, 2017). Currently, no international society has issued guidelines on the treatment of migraine comorbid with depression yet. Few medications are proven to target both disorders, and therefore, they might be used in their clinical management. Among these, venlafaxine and amitriptyline (Peck et al., 2015) provided the best evidence. Notwithstanding, new promising approaches, such as repetitive transcranial magnetic stimulation (rTMS), are reporting encouraging results in either condition (Leung et al., 2020).

#### Bipolar Disorders and Migraine

Up to 55% of migraineurs are also diagnosed with BD (Dresler et al., 2019). Such prevalence is particularly relevant in patients with type II BD (Low et al., 2003), with headache usually preceding the onset of manic episodes (Ortiz et al., 2010). This association seems to be bidirectional, as one third of patients with BD suffer from migraine (Leo and Singh, 2016). Treatment-wise, evidence has shown multiple therapeutic choices to be effective in either disorder, such as valproate and topiramate when stabilizing manic episodes and lamotrigine when targeting both bipolar depression and migraine (Vikelis and Rapoport, 2010).

#### Anxiety Disorders and Migraine

It is well-known that migraine has an up to 10-fold likelihood to be comorbid with anxiety disorders, especially generalized anxiety disorder (GAD) and panic disorder (PD) (Dresler et al., 2019). Indeed, it is not surprising that the prevalence of anxiety increases as headache episodes increase (Zwart et al., 2003). This is also true from a time perspective, as people with PD and migraine are proven to experience panic attacks earlier than non-migraineurs (Yamada et al., 2011).

The management of anxiety disorders comorbid with migraine mostly relies on antiepileptics, with topiramate, lamotrigine, and pregabalin being the best therapeutic options (Van Ameringen et al., 2004; Calandre et al., 2010; Casucci et al., 2010).

#### Obsessive-Compulsive Disorders and Migraine

Evidence shows a correlation between CM and obsessive-compulsive disorder (OCD), whose presence might influence migraine response to treatment, in both the short and long run (Buse et al., 2013). A previous study highlights how obsessive fearful thoughts about headache pain may fill patients' life more than migraine attacks (Curone et al., 2014). Furthermore, a consistently worse response to treatment was found to be more prevalent in chronic migraineurs with obsessive-compulsive traits as well as the tendency to undergo an early relapse (Curone et al., 2012).

#### Post-traumatic Stress Disorders and Migraine

In the last decades, evidence about the comorbidity of PTSD and migraine is grown. Up to 25% of migraineurs has PTSD, with higher rates than the general population (up to 10%). PTSD occurs with a higher prevalence in people suffering from CM (43%) than those with EM (9%) (Peterlin et al., 2008). This comorbidity is up to three times more common among women than men (Peterlin et al., 2011). Shared pathophysiological aspects, as the different hormonal maturation trajectory and the exposure to major psychological trauma, may explain the difference in comorbidity distribution among genders.

Recently, the 11th revision of the International Classification of Diseases (ICD-11) [World Health Organization (WHO), https://icd.who.int/en] has introduced the diagnosis of complex PTSD (cPTSD). It develops from prolonged interpersonal traumatic experiences without the opportunity to avoid them. Along with typical PTSD clinical dimensions, cPTSD has “disturbances in self-organization” (DSO; affect dysregulation, negative self-concept, and disturbances in relationships). This syndrome has a higher level of depression and dissociation and is more associated with medical diseases (Longo et al., 2019; Ho et al., 2021). Because, to date, data on the association between cPTSD and migraine are scarce, future studies will need to clarify the prevalence of this comorbidity.

#### Substance Use Disorders and Migraine

Data on the co-presence of migraine and substance use disorder (SUD) are slightly controversial. For instance, previous evidence suggests a lower prevalence of alcohol consumption/addiction among patients with migraine. This is possible since wine, beers, or spirits are commonly perceived as easy triggers for headache attacks (Zlotnik et al., 2014; Pellegrino et al., 2018). On the other hand, evidence on caffeine addiction points out how patients with CM were more likely to be frequent caffeine consumers than healthy individuals (Scher et al., 2004).

The literature shows that the association between migraine and substance abuse is no longer significant when controlling for PTSD and depression variables (Buse et al., 2013). For these reasons, substance abuse has to be considered as secondary to additional psychiatric comorbidities rather than migraine (Radat and Swendsen, 2005).

#### Somatic Symptom Disorders and Migraine

Conflicting results are also reported on the association of migraine and somatic symptom disorder. Previous studies have emphasized an equal prevalence of somatic symptoms among episodic migraineurs when compared with non-migraineurs (Lake et al., 2005). On the other hand, a higher prevalence of somatoform disorders was found among patients diagnosed with CM, with a direct association between somatic symptom severity and migraine frequency (Maizels and Burchette, 2004). Consistent with these results, children with migraine are found to display a higher set of somatic complaints (Bruijn et al., 2010), with a heavier sense of shame and fear than their healthy counterparts (Tarantino et al., 2015).

### Supposed Pathophysiological Mechanisms

The elevated rates of comorbidity between psychiatric disorders and migraine indicate that pathophysiology of these disorders may share several, common mechanisms. Some of these commonalities are listed below. Although they have been reported separately, some authors speculate that different pathophysiological mechanisms, which would explain the comorbidity between psychiatric disorders and migraine, may overlap and intersect with each other.

#### Neurotransmitters and Psychiatric Comorbidity

As previously reported in the literature, migraineurs, like depressed patients, show altered serotonin blood levels, i.e., higher during migraine attacks and significantly lower between them. These neurochemical alterations would favor an unbalance activity of the brainstem nuclei, a condition that may predispose to the activation of the trigeminovascular nociceptive pathway and may favor the abnormal neuro-vascular coupling accompanying CSD as well (Hamel, 2007). In addition, migraine might be treated with drugs acting on the serotonin system, such as triptans as painkillers, tricyclic antidepressants, and selective serotonin reuptake inhibitors (Silberstein et al., 2012). Other monoamines may be involved in the mechanisms of psychiatric bidirectional comorbidity with migraine. A study showed how a specific dopamine D2 receptor allele is present in migraineurs comorbid with aura, anxiety, and depression (Peroutka et al., 1998). Moreover, depressed chronic migraineurs have significantly lower GABA cerebrospinal fluid levels than non-depressed patients. This may suggest that also this neurotransmitter may play a key role in the pathophysiology of such comorbidity (Vieira et al., 2006).

#### Neuroinflammation and Psychiatric Comorbidity

The neuro-inflammation hypothesis has always been considered in the etiology of MDD and migraine. Indeed, evidence of altered hypothalamic–pituitary adrenal (HPA) axis was found in both disorders (Peres et al., 2001; Gonda et al., 2019). Obese patients with CM and depression showed significative higher pro-inflammatory cytokine blood levels, suggesting a link between these conditions (Bigal et al., 2007). Similarly, a common neuro-inflammatory diathesis might be seen both in migraineurs and in patients with BD. Pro-inflammatory cytokines like TNF-α and IL-1, in fact, may take part in the comorbidity process (Brietzke et al., 2012).

#### Genetics and Psychiatric Comorbidity

Previous studies have shown that migraine and MDD are bound by a bidirectional red thread, meaning that migraine might cause or be the cause of MDD (Moschiano et al., 2011). Indeed, some authors have suggested how these two conditions show a shared set of genes, especially when they are comorbid together (Schur et al., 2009; Ligthart et al., 2014).

As for MDD, also for BD, a common inheritance with migraine might be assumed. Genome-wide association studies, in fact, have highlighted a shared set of single-nucleotide polymorphisms (SNPs) encompassing a region of the gene KIAA0564 (Oedegaard et al., 2010). The gene KIAA0564 has putative ATPase activity expressed in the brain, as seen in patients with FHM2, and one transcript of this gene shows a pattern of expression in the whole brain, substantia nigra, amygdala, and hypothalamus, all regions known to be involved in both migraine and BD (Oedegaard et al., 2010). Evidence has shown that many neurotransmitters seems to be involved in the comorbidity of both disorders, such as serotonin (Mahmood and Silverstone, 2001; Hamel, 2007), glutamate (Vaccaro et al., 2007; Chen et al., 2010), and dopamine (Akerman and Goadsby, 2007; Ashok et al., 2017). It was also reported in literature that patients with migraine and BD share mutations on calcium and sodium channels, explaining why they both may respond to anticonvulsants like sodium valproate (Askland et al., 2009; de Vries et al., 2009).

#### Stress and Psychiatric Comorbidity

Stressful events predispose, trigger, or worsen psychiatric disorders. For example, PTSD is a consequence of major psychological trauma, and traditionally, MDD could be classified as reactive or endogenous if a significant life stressor is present before the onset of the symptoms or not. Similarly, stressful events characterize the clinical presentation of migraine and mark migraineurs' life. Stress and migraine share mutual characteristics, as the first might be considered as a trigger for migraine episodes and, conversely, the second is a well-established source of stress (Dresler et al., 2019). The process of central sensitization, commonly claimed to be at the base of migraine evolution to a chronic form, postulates disrupted processing taking place in the trigeminal nucleus caudalis for pain and in limbic structures, such as the amygdala and insula, for stress- and anxiety-related disorders (Grassini and Nordin, 2017). Some researchers differently postulate that patients in which a failure of limbic structures in adjusting to pain may occur, therefore resulting in an abnormal endocrine response that in turn leads to an altered response to stress, may belong to a “limbically augmented pain syndrome” (Rome and Rome, 2000).

#### Neurocircuits and Psychiatric Comorbidity

From a neurophysiological point of view, a dysfunctional neurolimbic network (Schwedt et al., 2013) might explain this aberrant interaction of pain and mood and therefore can support the clinical connection between migraine and depressive and anxiety disorders (Maizels et al., 2012).

Indirect evidence of this shared pathophysiological mechanism in the neurolimbic network is provided by a recent retrospective study in patients with comorbid migraine and unipolar depression treated with a therapeutic paradigm of high-frequency rTMS (HF-rTMS) over the left dorsolateral pre-frontal cortex (l-DLPFC) (Kumar et al., 2018). In addition to the clinical improvement of depression, a decrease in frequency, severity, and functional disability of migraine was reported. These findings may be explained with a sustained modulation effect, generated by the HF-rTMS therapeutic paradigm, of l-DLPFC, an area involved in cognitive control of pain.

## Comorbidity With Other Pain Syndromes

### Scientific Evidence of Comorbidity

Migraine patients often experience pain outside the territories primarily involved, as those innervated by the trigeminal nerve. The first cervical roots, C1–C3, have an anatomical and functional contingencies with the trigeminal nucleus caudalis, so migraine attack is usually diffused into the neck (Vincent, 2011). Besides, migraine pain allows the spread of allodynia phenomenon in the shoulder and even upper limbs (Burstein et al., 2000b). Central sensitization occurs during single attacks, so migraine patients are prone to comorbidities with other pain syndromes sharing this phenomenon as the main causal factor (de Tommaso et al., 2016; Arendt-Nielsen et al., 2018).

A bidirectional association has been observed between migraine and other, often chronic pains such as chronic low-back pain (Ashina et al., 2018), which accompanies dysmenorrhea (Miller et al., 2018; Gagnon and Elgendy, 2020), and temporomandibular disorder (Grossi et al., 2009). Results of the German Headache Consortium study showed that the OR of having frequent low-back pain was between 2.1 (95% CI: 1.7–2.6) and 2.7 (95% CI: 2.3–3.2) times higher in all episodic headache, including migraine, and between 13.7 (95% CI: 7.4–25.3) and 18.3 (95% CI: 11.9–28.0) times higher in all patients with chronic headache subtypes when compared with non-affected subjects (Yoon et al., 2013). But in recent years, much attention has been given to fibromyalgia (FM), as it seems to be strongly associated with migraine, especially if chronic. FM is a chronic and disabling disease dominated by diffuse pain and several associated symptoms, such as sleep disorder, cognitive impairment, and fatigue (Wolfe et al., 2010).

In the last 10 years, many studies confirmed a high prevalence of FM among patients with migraine, varying from 5% to more than 30%, depending upon the type of population considered (de Tommaso, 2012; Küçükşen et al., 2013). A high prevalence of FM was found in tertiary headache centers, where patients with severe migraine are prevalently followed up. FM comorbidity seems to be a hallmark for severe migraine, characterized by frequent headache, general disability, allodynia, and sleep disturbances (de Tommaso, 2012; de Tommaso et al., 2016).

Patients with FM suffer from CM and chronic TTH, while EM, and especially migraine forms with very sporadic attacks, like migraine with pure visual aura, rarely shares this comorbidity (de Tommaso, 2012). Factors favoring evolution into CM, such as sleep disturbances, prevail among CM with FM comorbidity (de Tommaso et al., 2014a).

FM comorbidity would not be a feature of a long history of migraine, as cases of FM are present even among migraine child cohorts (Kashikar-Zuck et al., 2013; de Tommaso et al., 2017). In child cohorts, the comorbidity with FM defines a clinical phenotype with more severe migraine, higher anxiety and depressive symptoms, and lower quality of life in all domains (Kashikar-Zuck et al., 2013).

Detecting FM in migraine patients could help in individuating patients with a profile of severe illness and poor quality of life. Clinical trials in FM patients displayed a low efficacy profile, with several adverse events and a prominent nocebo effect (Häuser et al., 2012). The correct therapeutic approach to single causes of comorbidity in such complicated patients could improve their global clinical picture (Affaitati et al., 2020). A recent observational study on the effects of preventive treatments after 3 months of therapy showed that patients with FM have a profile of resistance to first-line preventive drugs (Delussi et al., 2020). Tricyclic antidepressant amitriptyline was actually the most frequently prescribed drug for the treatment of migraine patients comorbid with FM (Delussi et al., 2020). However, researchers did not report the possible effect of amitriptyline on specific symptoms of FM and how the mild improvement of migraine could impact the disability linked to diffuse pain (Affaitati et al., 2020). Another important topic could be the assessment of the effects of therapeutic approaches to severe migraine, like botulinum toxin (Diener et al., 2010) and CGRP monoclonal antibodies (Edvinsson, 2019), to the global clinical impairment of FM.

### Pathophysiological Basis of Comorbidity

FM is one of the most diffuse and disabling conditions sharing with primary headaches central sensitization as the main pathophysiological mechanism (Arendt-Nielsen et al., 2018). Migraine and TTH are included into the central sensitization-related syndromes, which often coexist in a complex modality. The recent classification of chronic pain has included the category of “nociplastic pain” specifically referring to pain that “arises from altered nociception despite no clear evidence of actual or threatened tissue damage” (IASP Terminology, 2020). Central sensitization implies hyper-function of neurons and circuits in nociceptive pathways with increased neurons excitability and synaptic efficacy as well as reduced inhibition. It is mainly based on the remarkable plasticity of the somatosensory nervous system in response to different causes, neural-inflammation, or neuronal damage (Latremoliere and Woolf, 2009). In migraine, the inflammation occurring at the perivascular and meningeal level is followed by sensitization of second-order nociceptive neurons and wide dynamic range neurons within the trigeminal caudal nuclei, and third-order nociceptive neurons within the thalamus, with hyperalgesia and allodynia involving the skin and the muscles in the head, neck, and other somatic sides (Burstein et al., 2000b). In FM, the initial causes of pain are sometimes unknown and sometimes are due to inflammation or trauma. Moreover, a hyperactivity of cortical regions devoted to pain processing has been demonstrated by neuroimaging studies in FM (López-Solà et al., 2017) and migraine (Moulton et al., 2011). Neurophysiological studies based on bioelectrical correlates of nociceptive and multimodal stimuli stated that phenomena of reduced habituation to repetitive stimuli, especially the painful ones, accompany central sensitization phenomena in both FM and migraine (Coppola et al., 2013; Choi et al., 2016).

More recent studies underlined the presence of small fiber pathology in patients with FM (Oaklander and Nolano, 2019). In more than 50% of FM patients, a proximal partial loss of skin sensitive terminals has been detected. These FM cohorts with small fibers involvement include patients with migraine (Vecchio et al., 2020). The FM subgroup with migraine comorbidity did not show different neurophysiological and skin biopsy features except for a trend toward a more expressed lack of habituation to repetitive painful stimuli (de Tommaso et al., 2014b).

In FM patients, including those with associated migraine, proximal skin denervation corresponded to reduced habituation of laser-evoked responses (Vecchio et al., 2020). The occurrence of this phenomenon is in agreement with the hypothesis that the loss of cortical adaptation to peripheral inputs could be related to an initial condition of hypo-activation followed by a delayed response potentiation (Coppola et al., 2013).

The evidence of a mild small fiber pathology in migraine patients with FM comorbidity opens a new scenario about the causes of the coexistence of peripheral and central nervous system (CNS) dysfunction, like genetic abnormalities of voltage-gated sodium channels (Eijkelkamp et al., 2012).

Very pertinent to the pathophysiology migraine is the observation that a mechanism involving the release of CGRP was also described for pain in musculoskeletal disorders and may be a direct cause of pain in other conditions. Musculoskeletal tissues are rich in CGRP-immunoreactive nerves and are associated with altered CGRP expression pain. These observations paved the way for randomized controlled trials of monoclonal antibodies for the treatment of pain conditions other than migraine (Walsh and McWilliams, 2019).

## Sleep-Related Disorders and Migraine

### Scientific Evidence of Comorbidity

The relationship between sleep and migraine has always been known, but current knowledge on the exact nature of the link between migraine and sleep remains incomplete and unclear. A large amount of epidemiological data shows a high comorbidity between migraine and sleep disorders (Drake et al., 1990; Sahota and Dexter, 1990; Dodick et al., 2003; Olesen et al., 2006).

#### Migraine and Insomnia

The association between migraine and insomnia is statistically significant, since one presents a risk of incidence, if the other condition is present, equal to about twice (OR = 1.4–2.6) the risk of incidence of only one of the two conditions (Uhlig et al., 2014). This relationship is bidirectional, and the association is stronger in more frequent, severe, or comorbid headache (Ødegård et al., 2013). Considering migraine sufferers only, a reduced sleep duration (<6 h per day) is independently associated with an increase headache attack frequency (Song et al., 2018). Although migraineurs suffer more frequently from disorders, the average sleep duration does not differ between migraineurs and non-migraineurs (Song et al., 2018). On the contrary, a reduced “sleep quality” (a satisfaction index based on the evaluation of how restful sleep is) is significantly more frequent in migraine sufferers (Song et al., 2018). The prevalence of insufficient sleep is statistically higher in migraine sufferers than in subjects with other forms of headache and then in subjects without headaches. Multivariate analysis confirms an OR (corrected for sociodemographic variables, anxiety, and depression) of 1.8 for migraine in subjects with insufficient sleep (Kim et al., 2017). Neurophysiological data support the hypothesis that relative sleep deprivation and varying robustness of the neurobiological arousal system may be among several causal factors for a migraine attack (Engstrøm et al., 2014; Rains, 2018).

#### Migraine and Sleep-Disordered Breathing

Since the first systematic descriptions, it was not clear whether morning headache and sleep apnea headache were two distinct nosological entities. Similarly, it was debated if the awakening headache was the recurrent manifestation of a primary headache, such as migraine. The first study of 304 patients concludes that morning headache is not an integral part of obstructive sleep apnea syndrome (OSAS) (Aldrich and Chauncey, 1990). Based on the results of many studies, morning headache does not have strictly specific characteristics (Loh et al., 1999; Neau et al., 2002; Alberti et al., 2005). Overall, there are also conflicting literature data on the association between morning headache and OSAS severity (Aldrich and Chauncey, 1990; Loh et al., 1999; Greenough et al., 2002; Neau et al., 2002; Göder et al., 2003; Alberti et al., 2005), but comorbidity between OSAS and migraine was not considered in most studies. Treatment of the respiratory disorder results in an improvement of the morning headache. Other studies say that such improvement, as well as headache, is likely to be non-specific (Aldrich and Chauncey, 1990; Poceta and Dalessio, 1995; Paiva et al., 1997; Loh et al., 1999; Göder et al., 2003; Ohayon, 2004). Considering specific forms of primary headache, the cumulative incidence of migraine was significantly higher in a large sleep-disordered breathing (SBD) cohort than in the comparison cohort (Harnod et al., 2015). The prevalence of primary headache in OSAS varies from 11 to 25% up to more than 40% (Loh et al., 1999). Habitual snoring was more frequent in chronic daily headache subjects (24%) than in controls (14%) (Scher et al., 2003a). However, the wide discrepancy in reported headache prevalence may reflect differences in study design (retrospective or prospective), in the definition of the headache itself and in considering patients who are undergoing polysomnography with suspicion of OSAS. Several studies have shown the effectiveness of continuous positive airway pressure (CPAP) treatment in improving all types of headache. These studies highlight that even patients with a mild form of OSAS improve with non-invasive ventilation. These data suggest treating headache patients with OSA symptoms, with any degree of severity (Johnson et al., 2013).

#### Migraine and Restless Legs Syndrome

Both clinic-based (Young et al., 2003; Rhode et al., 2007; d'Onofrio et al., 2008; Chen P. K. et al., 2010; Suzuki et al., 2011; Lucchesi et al., 2012; Lin et al., 2016; Valente et al., 2017) and large-scale population-based studies suggest an association between migraine and restless legs syndrome (RLS) (Schürks et al., 2012; Winter et al., 2013). The association was confirmed also after adjustment for confounding factors such as age, sex, major depression, anxiety, and sleep quality (Zanigni et al., 2014). RLS also accounts for poorer sleep quality in those patients with comorbidity (Valente et al., 2017). The frequency of migraine attacks correlates positively with the prevalence of RLS, and the MA had a stronger trend of association with RLS (Lin et al., 2016). The authors suggest that, at least in part, this relationship might be explained by a pharmacological overload of serotoninergic drugs, which might interfere with the physiological balance between dopaminergic and serotoninergic pathways (Valente et al., 2017).

#### Migraine and Narcolepsy

Some studies showed an increased frequency of migraine (37–54%) in patients with narcolepsy (Dahmen et al., 1999, 2003). However, a large multicenter observational study found an increased frequency of TTH (60.3 vs. 40.7%) but not migraine (21.9 vs. 19.8%) in narcolepsy patients compared with controls (Evers, 2003). More recent data confirm that patients with narcolepsy and idiopathic hypersomnia more frequently experienced headache than the healthy controls and that the patients with both conditions more commonly experienced excessive daytime sleepiness and had reduced total sleep time than the patients with narcolepsy without headache (Suzuki et al., 2015).

#### Migraine and Advanced Sleep Phase

Although there is no robust epidemiological evidence, the description of a family with a genetic mutation related to a condition characterized by the so-called advancement of the familial advanced sleep phase (FASP) (Xu et al., 2005) inspired the hypothesis of a close physiological correlation between migraine and this sleep disorder as well as a brilliant editorial (Ahn and Goadsby, 2013). These data give the opportunity to assume the important role of the hypothalamus in migraine pathophysiological mechanisms and hypothesize any new therapeutic targets (Ahn and Goadsby, 2013).

#### Migraine and Parasomnias

Numerous old studies have shown the association between migraine and sleepwalking (Barabas et al., 1983; Giroud et al., 1986; Pradalier et al., 1987). Other studies have shown the high prevalence of various parasomnias (pavor, sleepwalking, and enuresis) even in adults (in the first two decades of life) (Messina A. et al., 2018). In several studies, subjects with bruxism seem to have a high prevalence of primary headaches and especially CM (Dexter, 1979; De Luca Canto et al., 2014). The serotonergic circuits of the median raphe nucleus have been involved as a common key structure between migraine and parasomnias, as they play a central role in pain processing and in the determination of sleep/wake rhythms (Messina A. et al., 2018).

#### Sleep-Related Migraine

The International Classification of Headache Disorders, 3rd edition (ICHD, 2018) does not include forms of sleep-related headache or sleep-related migraine; however, some migraine patients have >50 or >75% of sleep-onset migraine attacks (Della Marca et al., 2006b; Rains, 2018). The chronobiological mechanisms are likely more involved in specific forms of headache. In this view, some authors suggest that data on this form of migraine should be collected (Rains, 2018).

#### Migraine, Sleep, and Chronification

All types of sleep dysregulation are involved in the chronicity mechanisms of primary headaches.

Every year, up to 3% of patients (Scher et al., 2003b) may experience the progression of EM into a chronic form (Rains, 2008). The potential mechanisms of chronification are manifold, and sleep disturbances have been identified among the risk factors associated with chronic headaches. Others are overuse of drugs, stress, psychiatric disorders, and obesity (Rains, 2008). Consequently, screening and treatment of sleep disorders are recommended in the clinical management of migraine (Poceta and Dalessio, 1995; Ong and Park, 2012).

### Supposed Pathophysiological Mechanisms

Migraine and sleep disorders have a high prevalence in the general population but are extremely and so overlapped that it is difficult to believe that their comorbidity is only incidental.

In addition to the pure epidemiological evidence, other physiological aspects strongly suggest close pathophysiological links between migraine and sleep fluctuations: circadian oscillations in the sleep/wake rhythm (cyclic biological changes that occur in the 24-h interval) (Ahn and Goadsby, 2013), changes in the ultradian rhythm [shorter than a day, the alternation of non-rapid eye movement (NREM)/REM phases in sleep cycles] (Jennum and Jensen, 2002), and modifications of the arousal mechanisms (Bruni et al., 2004; Della Marca et al., 2006a).

Moreover, key structures have an unequivocal modulatory involvement in both migraine and sleep, namely, the hypothalamus, brainstem (Goadsby, 2005), and thalamus–cortical circuits (Coppola et al., 2016).

Finally, orexinergic (Hoffmann et al., 2015), serotoninergic (Goadsby et al., 2017), and dopaminergic (Charbit et al., 2010) neurotransmissions have a crucial and common role in migraine and sleep. Interestingly, premonitory symptoms of migraine such as yawning, craving for food, and gastrointestinal disturbances, supposed to be dopamine-mediated (Akerman and Goadsby, 2007), were more frequently reported in migraine patients with RLS compared with those without RLS (Cologno et al., 2008). It is well-known that migraine is characterized by a hypersensitivity to dopamine (Sicuteri, 1976; D'Andrea et al., 2006) and that dopaminergic projections play an important role in the processing of trigeminovascular information (Charbit et al., 2010). Some authors have suggested that a dysfunction of the hypothalamic dopaminergic nucleus A11 may be part of the complex pathophysiology of migraine and RLS and that both disorders have a common genetic basis, also involving dopaminergic transmission (Bonati et al., 2003; Charbit et al., 2010).

Patients with migraine do not differ from non-migraineurs in sleep macrostructure but have a marked reduction in the polysomnographic parameters of arousal in NREM sleep and a lower incidence of “cortical” arousals in REM sleep (hypo-arousability) than do non-migraineurs (Della Marca et al., 2006b). On the other hand, migraineurs showed an increased instability of the autonomic balance during sleep (Vollono et al., 2013).

In conclusion, since the most reproducible hypnological marker in migraine is hypo-arousability (Bruni et al., 2004; Della Marca et al., 2006b; Vollono et al., 2013; Engstrøm et al., 2014; Rains, 2018), it is possible to hypothesize that the dysfunction of arousal system is the expression of the modified brain's ability to process exogenous and endogenous stimuli during sleep.

## Gastrointestinal Disorders and Migraine

Due to an overly complex multifactorial pathway, gastrointestinal disorders are quite common among migraine patients. In fact, on the one hand, it is well-known that there is a higher prevalence of migraine in people with much reflux symptoms, diarrhea, constipation, or nausea than in those without them (Aamodt et al., 2008). On the other hand, nausea and vomit are common symptoms of the migraine attack, according to classifying criteria (ICHD, 2018); and alterations of the intestinal transit (leading to constipation or diarrhea) are part of the autonomic symptoms accompanying pre- and post-dromal phases of the attack (Gazerani and Cairns, 2018). The gastrointestinal comorbidities in patients with migraine involve disorders in different organs of gastrointestinal (GI) tract, from the mouth to the bowel.

### Scientific Evidence of Comorbidity

A recent multicenter study evidenced that the presence of periodontitis (a serious gum inflammatory condition due to bacterial infections) is independently related to CM, with a higher prevalence than patients with EM (53.9 vs. 44.6%) (Leira et al., 2020).

In a large questionnaire-based cross-sectional study (the Head-HUNT Study), researchers observed that the more severe the gastroesophageal reflux disease (GERD), the more prevalent is migraineur and non-migraineur headache (Aamodt et al., 2008). A similar association between the presence of GERD and its severity and headache was also evidenced in other two large studies (Saberi-Firoozi, 2007; Katić et al., 2009). More recently, a more detailed analysis was performed among patients with dyspepsia. Fifty-four percent of patients with epigastric pain syndrome also suffered from migraine, but headache seems to be not induced by meal ingestion. Besides, migraine prevalence in patients with postprandial distress syndrome was 76%, and almost all patients reported a meal-related headache with a correlation between the entity of the gastric discomfort threshold and migraine severity (Di Stefano et al., 2019).

*Helicobacter pylori* is the bacteria responsible for gastric ulcer and its neoplastic degeneration, and its infection seems to negatively influence migraine symptoms, according to the patient's ethnicity, the place of residence, and the bacterial strains (Cámara-Lemarroy et al., 2016). It was also observed that its infection is more prevalent in patients with migraine than in controls (Su et al., 2014), and the bacterial eradication is related to relief of migraine symptoms (Faraji et al., 2012; Savi et al., 2014).

Abdominal discomfort ascribable to the liver is almost double in patients with migraine than in controls (Kurth et al., 2006). Particularly, the clinical presentation of hepatobiliary disorders seems to be severer in patients with migraine (Aggarwal and Bielefeldt, 2013) and related to it by a common genetic background, as suggested by a large study on twins (Nilsson et al., 2010). Also, non-alcoholic fatty liver disease was related to headache in general (with a borderline value for the significance in patients with migraine) (Martami et al., 2018) and MA in particular (Celikbilek et al., 2014).

Celiac disease (CD) is a genetically based autoimmune systemic disorder triggered by gluten (a cereal grain group of protein) ingestion and characterized by GI and non-GI symptoms, including migraine (Taylor et al., 2016). A recent meta-analysis reported that CD and headache (mainly migraine) have a bidirectional relationship, and it was suggested to screen headache patients for CD since they may benefit from a gluten-free diet (GFD) (Zis et al., 2018). The GFD improves migraine in patients with CD (Ameghino et al., 2019), but among migraineurs, the CD is present only in 2.4% of subjects (Zis et al., 2018), so only a limited number of patients deserves screening for CD. It should be advised only to patients with an important presence of GI symptoms and/or several non-GI symptoms (Taylor et al., 2016), also because the beneficial effect of GFD in non-celiac patients with migraine is not clear (Beuthin et al., 2020).

Irritable bowel syndrome (IBS) and migraine are often comorbid, and researchers observed that the longer the history and severity of migraine, the higher the risk of being affected by IBS (Li et al., 2017); moreover, in case of co-occurrence of both disorders, patients are more prone to develop more complicated clinical pictures (Georgescu et al., 2018). IBS and migraine share several features: both are chronic disorders, diagnosed only by symptomatic criteria (standardized diagnostic biomarkers are not available), characterized by recurrent pain attacks, more prevalent among females, and comorbid with somatic (interstitial cystitis, FM, and chronic fatigue syndrome) and psychiatric (abuse behavior, insomnia, anxiety, and depression) diseases (Georgescu et al., 2018).

Compared with that in the general population, migraine is more prevalent also in patients with inflammatory bowel disease (IBD), in both adults (Moisset et al., 2017) and children (Ben-Or et al., 2015), being their most prevalent neurological disorders (Oliveira et al., 2008). IBD includes Crohn's disease and ulcerative colitis, both characterized by relapsing/remitting acute inflammations.

Migraine seems to be more prevalent among patients with constipation (Aamodt et al., 2008), and it has been proposed that the dietary treatment for this GI complaint leads to migraine improvement (Prakash and Mullen, 2010). Also, laxative treatments seem to be useful to improve migraine-related disability and severity in children with migraine and constipation (Rezaeiashtiani et al., 2019). On the other hand, constipation is more prevalent in patients with migraine than in those with TTH and non-headache subjects (Martami et al., 2018).

### Supposed Pathophysiological Mechanisms

Attempting to speculate about the pathophysiological bases of GI comorbidities in patients with migraine, we can invoke three main different mechanisms of action: the involvement of the enteric and autonomic nervous system (ENS and ANS), the production of inflammatory cytokines, and dysbiosis, that is, a microbial imbalance or maladaptation.

During embryogenesis, the ENS develops simultaneously with the CNS, and they are connected by the modulation of the vagal nerve. Therefore, although it is unclear if the correlation between migraine and gastric digestive symptoms is due to a primary neurologic or gastric issue, gastric symptoms can be regarded as part of the spectrum of dysautonomia dysfunctions related to a migraine attack. To support this hypothesis, the entity of gastroparesis is related to the severity of migraine intensity (Boyle et al., 1990), and negative gastroscopic results are observed in 90% of patients with migraine who complained of gastric symptoms (Meucci et al., 2005). On the other hand, a bidirectional connection between CNS and ANS/ENS is suggested by some reports of migraine improvement after the pharmacological treatment of gastric symptoms (Mavromichalis et al., 1995; Spierings, 2002; Hwang et al., 2016). Nevertheless, the widely used proton-pump inhibitors are regarded as a worsening factor for migraine (Makunts et al., 2019), meaning that not the drugs' mechanism of action but the relief of GI symptoms leads to migraine improvement. The involvement of ENS/ANS was also called into question IBS comorbidity. Although sexual hormones, genetics, and biopsychosocial background seem to underpin the comorbidity, ANS was theorized as the link with the shared central sensitization and allodynia during the acute attack onset (Chang and Lu, 2013). Lastly, ANS/ENS dysfunctions, together with the use of anticholinergic drugs to prevent migraine, dehydration, and an inadequate dietetic regimen, were supposed to be at the base of constipation comorbidity (Diaz et al., 2020).

Inflammatory cytokines seem to be potentially involved in the inflammation-accompanied IBD and other GI comorbidities. The increase in pro-inflammatory substances such as serum pentraxin 3, soluble TNF-like weak inducer of apoptosis (Leira et al., 2018), and serum procalcitonin (Gonzalez et al., 2016) was found in case of periodontitis. The release of different inflammatory cytokines by Helicobacter Pylori (HBP) infection may contribute to explain its comorbidity with migraine (Arzani et al., 2020). It is also possible to hypothesize a role for the cholecystokinin (CCK), a duodenal endocrine peptide that is involved in gallbladder movement, lipid digestion, and hunger suppression, which also has vasoactive activity and coexists with CGRP in trigeminal ganglion (O'Connor and Van der Kooy, 1988; Ruiz-Gayo et al., 2006). In turn, the CGRP, by modulation of vagal parasympathetic outflow (Li et al., 1998), is involved in the pathophysiology of gallstone disease (Mulvihill and Yan, 1995). Nonetheless, CGRP is a key peptide in response to GI inflammation (Holzer, 2007), and its secretion from peripheral sensory nerves could have CNS consequences by sustaining a pro-inflammatory permissive state, which in turn may lower the threshold to the onset of migraine attacks. Finally, we can hypothesize that also constipation can induce gut inflammation and permeability, with reabsorption of molecules that can trigger migraine attacks. This is the case of the lipopolysaccharide that, in animal models, can induce neuroinflammation in trigeminal ganglia (Kemper et al., 1998) and was adopted as an experimental model of migraine attack (Fiebich et al., 2002).

It is well-known that the microbiota and brain functions are related by a reciprocal modulation: several neuropsychiatric disorders have been associated with impaired microbiota (Tremblay et al., 2021). Interestingly, in animal models, gut microbiota dysbiosis contributes to chronicity of migraine-like pain by upregulating TNF-α level in the trigeminal nociceptive system, while probiotic administration significantly inhibited the antibiotic-produced migraine-like pain prolongation (Tang et al., 2020). Despite this preclinical evidence, clinical studies in humans do not clearly support the efficacy of probiotics in treating patients with migraine (Dai et al., 2017).

## Immunological Disorders and Migraine

### Scientific Evidence of Comorbidity

The relationship between migraine and immunological/autoimmune diseases is overly complex and not completely defined, but several epidemiological, clinical, and laboratory evidence supports this association.

From an epidemiological point of view, it is widely accepted that migraine more commonly affect women than men (Lipton et al., 2001), and this is consistent with the high prevalence of autoimmune diseases in women (Pennell et al., 2012).

Sometimes, headache and specifically migraine can be a clinical manifestation of many autoimmune disorders, either for those primarily involving the CNS, like multiple sclerosis (MS), or systemic disorders, like systemic lupus erythematosus (SLE). It is not clear whether the headache, and specifically migraine, is a direct, specific manifestation of disease and its activity or it is only a concomitant disorder; and, most importantly, it is a matter of debate if headache and in particular migraine can predispose some patients to the subsequent development of an autoimmune disorder.

Several studies assessed migraine prevalence in MS patients, which varies consistently among studies, ranging from 19.8 to 78% (Abb and Schaltenbrand, 1956; Poser et al., 1966; Clifford and Trotter, 1984; Freedman and Gray, 1989; D'Amico et al., 2004; Vacca et al., 2007; Boneschi et al., 2008; Nicoletti et al., 2008; Villani et al., 2008; Putzki et al., 2009; Kister et al., 2010; Möhrke et al., 2013). This variability can in part be attributed to the difference in the study design, populations included, and MS and migraine criteria adopted. A meta-analysis including eight studies (1,864 MS patients and 261,563 controls) found a significant association between migraine and MS with an OR = 2.60 (95% CI: 1.12–6.04); for MO with MS, OR was 2.29 (95% CI: 1.14–4.58), without a significant heterogeneity (Pakpoor et al., 2012). Interestingly, in a large population-based cohort (Nurses' Health Study II), a history of migraine was associated with an increased risk of developing MS (1.39 times higher), but the difference in absolute MS risk between migraineurs and non-migraineurs was small (Kister et al., 2012). When migraine occurrence was considered in relation to clinical characteristics and subtypes, MS patients with headaches and in particular with migraine are significantly younger, are more often female, and more frequently have a diagnosis of clinically isolated syndrome (CIS) or relapsing/remitting MS (RRMS) and lower Expanded Disability Status Scale score (EDSS) than MS patients without headaches (Möhrke et al., 2013). In contrast, headache with tension-type characteristics was more often reported by MS patients with a progressive form of the disease (D'Amico et al., 2004).

The relationship between migraine and the disease activity was investigated by a study of Tabby et al. (2013), who showed that MS patients with migraine presented more relapses than patients without migraine and that 85% of patients whose attacks were often or always of severe intensity reported a headache worsening during MS exacerbations. Furthermore, Kister et al. (2010) observed that migraine in MS patients was significantly associated with a more symptomatic course of the disease, but not with disability or T2 lesion burden on brain magnetic resonance imaging (MRI). Headache is indeed the most common indication for performing MRI in cohorts with radiologically isolated syndrome (RIS). Accordingly, headache was the reason for neuroimaging in about half of subjects from a case series collected by Granberg et al. (2013), but only a few of them showed a progression of MRI lesions during the next 2 to 5 years.

As far as the influence of disease-modifying treatment for MS on migraine course is concerned, it is widely recognized that IFN-beta exacerbates attacks in MS patients already suffering from migraine or induce a *de novo* migraine in patients who did not suffer from headache before (Nikfar et al., 2010; Patti et al., 2012; De Jong et al., 2017). Conversely, a significant reduction of migraine frequency in the MS patients switching from IFN-beta to natalizumab, irrespective of clinical variables such as fatigue, anxiety, depression, and MIDAS scores, was observed (Villani et al., 2012).

Altogether, the above evidence suggests that migraine is a relevant symptom in MS especially in the early stages of the disease. In some cases, a previous personal history of migraine can be recorded; in other cases, headache developed *de novo* in temporal relationship to the neurological symptoms leading to the diagnosis of CIS or MS or in the course of the disease particularly during a relapse or in relationship to IFN treatment.

Much research investigated headache occurrence in SLE patients because headache was indicated as a typical although not specific manifestation of CNS involvement in SLE. In particular, the SLE Disease Activity Index (SLEDAI) included lupus headache as a descriptor, defined as a severe, persistent headache with often migraine-like features and unresponsive to analgesic treatment (Bombardier et al., 1992). However, in a recent study conducted by Hanly et al. (2013), only 1.5% of patients specifically meet the criteria of lupus headache, as defined in SLEDAI. In addition, if present, headache was associated with other neuropsychiatric manifestations. Not surprisingly therefore, lupus headache was not included in the American College of Rheumatology definition of neuropsychiatric syndrome in SLE. When SLE was considered without a specific mention to an SLE CNS involvement, no difference in headache prevalence emerged between controls and SLE patients in a meta-analysis carried out by Mitsikostas et al. (2004). Several studies more specifically focusing on the prevalence of migraine in SLE patients found, like for MS patients, a large variability of results with percentages ranging from 7.9 to 52% (Isenberg et al., 1982; Markus and Hopkinson, 1992; Sfikakis et al., 1998; Fernández-Nebro et al., 1999; Ainiala et al., 2001; Glanz et al., 2001; Whitelaw et al., 2004; Lessa et al., 2006). Some authors also found a higher prevalence of MA in patients with SLE (Brandt and Lessell, 1978; Glanz et al., 2001), but these data were not confirmed by others (Vázquez-Cruz et al., 1990; Fernández-Nebro et al., 1999; Glanz et al., 2001; Lessa et al., 2006; Katsiari et al., 2011).

Headache and migraine were associated with antiphospholipid antibodies (aPLs) and beta2GPI antibody positivity other than Raynaud's phenomenon in two (Weder-Cisneros et al., 2004; Lessa et al., 2006) out of three studies (Sfikakis et al., 1998).

Finally, based on the available evidence, the possible link between SLE and migraine has not been clarified, and therefore, the occurrence of headache in SLE patients in most cases does not itself require further investigation. Migraine in these patients should be classified according to International Headache Society (IHS) criteria and, in general, managed according to the available treatment guidelines.

A variety of dated studies investigated the association between antiphospholipid syndrome (APS) and migraine, reporting a migraine prevalence ranging from 0 to 30% (Hogan et al., 1988; Hering et al., 1991; Iniguez et al., 1991; Robbins, 1991; Tietjen, 1992). More recently, the Euro-Phospholipid Project revealed a prevalence rate of migraine of 20% in APS patients (Cervera et al., 2009), with the onset of headache preceding one or two decades before the APS diagnosis (Hughes, 2010). Some authors recommended screening for aPL in patients known to have migraine or recurrent headaches since there may be a link between migraine and stroke in APS patients (Cuadrado and Sanna, 2003). However, although some research reported higher prevalence of aPL in migraineurs patients as compared with healthy controls (Briley et al., 1989; Iniguez et al., 1991), others failed to find an association (Verrotti et al., 2000; Williams et al., 2008; Meroni et al., 2014). Conflicting results were obtained about anticardiolipin antibody (aCL) positivity in migraine (Levine et al., 1987; Iniguez et al., 1991; Robbins, 1991; Hinse et al., 1993; Gallo et al., 1994; Tietjen et al., 1998; Verrotti et al., 2000).

Antibodies to PT have been reported to occur in 50–90% of patients with APS (Vlagea et al., 2013). Furthermore, in a recent study, migraine headaches have also been observed more frequently in patients with both aPS antibodies and Raynaud phenomenon (Kopytek et al., 2018). According to Sanna et al. (2006), headache associated with APS is often untreatable, poorly responding to analgesics and typically starts several years before the diagnosis of APS. In spite of that, heparin followed by long-term anticoagulation with warfarin, which is the cornerstone of APS treatment, induces a clear improvement or resolution of migraine in many cases (Asherson et al., 2007; King and Odette, 2012; Erkan et al., 2014).

Some studies demonstrated a significantly higher prevalence of migraine in patients with primary Sjögren's syndrome (pSS) than in normal subjects (Pal et al., 1989; Gökçay et al., 2008). Therefore, it was claimed that both migraine and dry eye could be a part of a common inflammatory process. However, further evidence denied this association (Tjensvoll et al., 2013). Interestingly, in a study by Morreale et al. (2014) involving 120 pSS patients, headache was the most common neurological complaint referred by the patients (46.9%) followed by cognitive (44.4%) and mood disorders (38.3%). The most frequently observed headache was MO. Interestingly, cutaneous allodynia, a sign of central sensitization, was referred by 31% of patients with headache, and particularly in migraine. Migraine occurrence was also significantly related to SSA antibodies, MR spectroscopy (MRS) alterations (reduction of NAA levels or decrease in NAA/Cr ratio), and hemodynamic dysfunction at ultrasonographic evaluations, but not to the presence of vasculitis brain lesions and/or macrovascular damage [such as white matter (WM) lesions and MS-like lesions]. In addition, the frequency of headache and alterations to MRS appeared to be higher in patients with Raynaud's phenomenon.

Among the other systemic autoimmune disorders, rheumatoid arthritis (RA) seems to be more prevalent in migraineurs than in non-migraineurs (Kalaydjian and Merikangas, 2008; Le et al., 2011). Moreover, one recent study showed also that patients with migraine were more likely to develop RA later in life. This temporal relationship may imply a causal link between migraine and RA.

Several studies have investigated this association between migraine and atopic diseases in both adult and child populations. In particular, a relationship between asthma and migraine-type headaches has been reported especially in females as well as a greater prevalence of hay fever, rhinitis, and dermatitis in migraineurs than in healthy non-atopic controls (Mortimer et al., 1993; Wilkinson et al., 1994; Davey et al., 2002; Ku et al., 2006; Özge et al., 2006; Aamodt et al., 2007; Tollefsen et al., 2008). Asthma has also been indicated as a risk factor for new-onset CM (Lee et al., 2013; Gryglas, 2016; Martin et al., 2016). In most of these studies, however, diagnosis of allergic disorders is not definitive and is solely based on medical history and to the presence of allergic or respiratory symptoms. The most recent findings on this topic concern the greater risk of migraine in atopic children (Wang et al., 2016). Furthermore, the risk shows a cumulative effect of more allergic diseases and more allergy-related health care (Wei et al., 2018). Children and adolescents with migraine were more likely to complain of persistent asthma, the latter being associated with higher frequency and more disabling migraine attacks. Interestingly, the history of anti-asthmatic or anti-allergic therapies was associated with a decreased risk of migraine, suggesting their potential role on the prevention of migraine occurrence in these patients (Aupiais et al., 2017). Concurrent with the above results, a lower “degree of atopy” has been related with less frequent and milder migraine headaches in younger patients while a higher degree with more frequent and disabling attacks. In these patients, the administration of immunotherapy induced a decrease in the frequency of migraine headache and associated disability (Martin et al., 2011).

### Supposed Pathophysiological Mechanisms

Dysfunction of the immunological system can be the common pathophysiological link between migraine and immunological diseases. Indeed, some immunological dysfunction has been suggested to play a role in migraine pathogenesis (Kemper et al., 2001; Bruno et al., 2007). Compared with that in healthy subjects, a significant increase in CD4+ and a decrease in CD8+ populations has been found in migraine patients, which was associated with a reduction in immunoregulatory CD4+CD25+ cell levels. These findings suggest a possible failure of self-recognition mechanisms in migraine patients, which could predispose them to immunological and specifically autoimmune disorders (Arumugam and Parthasarathy, 2016).

In pSS patients, pro-inflammation-mediated mechanisms and endothelial dysfunctions of the cerebral microcirculation could account for the comorbidity with migraine and Raynaud's phenomenon (Morreale et al., 2014). For this reason, headache with migraine features and Raynaud's phenomenon may be attributed to a sort of “autoimmune endotheliitis” directly inducing perivascular inflammation and a vasomotor dysfunction.

One of the possible explanations of the occurrence of migraine *de novo* or an exacerbation of a preexisting migraine is the location of MS lesions in strategic sites of the nociceptive pathways involved in the processing of head pain in migraine, such as midbrain/periaqueductal gray matter areas (Gee et al., 2005).

The association between RA and SLE and migraine has been related to a shared dysfunction of the serotonergic system (Zeller et al., 1983; Hamel, 2007; Wang et al., 2017). Platelet serotonin levels are significantly decreased in RA patients and are inversely related to clinical RA activity (Zeller et al., 1983). Nonetheless, the production of inflammatory cytokines, such as TNF-α, was inhibited by the serotonin (Cloëz-Tayarani et al., 2003) and during treatment with serotonin reuptake inhibitors (Sacre et al., 2010).

An increased production of platelet-activating factor and the release of vasoactive neuropeptides can play a role both in asthma pathogenesis (Wasserman, 1994) and in the induction of migraine attacks (Sarchielli et al., 2004). Interestingly, transient receptor potential cation channel subfamily V member1 channels, which co-localize with vasoactive peptide CGRP and are implicated in migraine pathophysiology, were found to be overexpressed in asthmatic mice, and their antagonists effectively suppressed inflammation (Li et al., 2019). Nonetheless, a common genetic denominator is not negligible since children have been demonstrated to be at higher risk of asthma if their parents have a history of migraine (Gürkan et al., 2000).

## Migraine Comorbidity as Judged by Neuroimaging Techniques

A good clinical history and neurological examination are sufficient to make a diagnosis of migraine and to evaluate the association with other medical conditions (Evans, 2019). Nowadays, diagnostic tests are recommended only if an abnormal neurological examination, red flags for secondary headaches, atypical features of migraine, or changes in migraine characteristics are present. However, diagnostic tests are often performed in clinical practice to reduce diagnostic uncertainty, to address the concerns of patients, or for medicolegal reasons (Evans et al., 2020).

Since the late 1980s, MRI studies have disclosed the presence of small, punctuate, regions of high-signal intensity involving the deep or periventricular WM in patients with migraine (Hougaard et al., 2014). Infarct-like lesions involving the cerebellum and deep brain structures have also been described in migraine patients with and without aura (Kruit et al., 2005; Bashir et al., 2013). An increased risk of WM hyperintensities (WMHs) is present even in pediatric patients with migraine (Mar et al., 2013; Rocca et al., 2014). Whether these findings are migraine-specific and what factors might influence their presence are still a matter of debate. The prevalence of WM alterations in migraine patients varies widely among the studies (Kruit et al., 2004; Hamedani et al., 2013; Hougaard et al., 2014). Discordant findings have been found regarding the association between the occurrence of WMHs and a higher migraine attack frequency, longer disease duration, the female gender, and presence of migraine aura (Kruit et al., 2010; Palm-Meinders et al., 2012; Bashir et al., 2013; Gaist et al., 2016). Results of longitudinal studies investigating the progression of WMHs in migraine patients are also inconsistent, probably due to the use of different methods of WMH evaluation and the inclusion of patients of different ages (Kurth et al., 2011; Hamedani et al., 2013; Mar et al., 2013). WM alterations are common in people aged 50 or over and in individuals with cardiovascular risk factors (e.g., hypertension, DM, or smoking) (Cannistraro et al., 2019). Some studies showed that migraine patients with cardiovascular risk factors have a higher risk of harboring WM abnormalities, suggesting that other potential etiologies rather than migraine might explain the presence of these alterations (Cooney et al., 1996; Bashir et al., 2013). An abnormal cerebrovascular reactivity leading to focal oligemia, atherosclerotic rick factors, endothelial dysfunction, and cardiac abnormalities, including PFO and atrial septal defect, are some of the mechanisms that might contribute to the occurrence of WM alterations in migraine patients (Bashir et al., 2013; Lee et al., 2019; Hoogeveen et al., 2020). Increased neuronal activation, neurogenic inflammation, and metabolic dysfunction have also been considered in the pathogenesis of WMHs in patients with migraine (Porter et al., 2005).

The imaging features of WMHs of migraine patients may resemble the WM lesions seen in patients with inflammatory diseases, like MS, representing a diagnostic challenge. The presence of cortical lesions or more than three periventricular lesions or the identification of an intralesional vein may provide important pieces of information in the diagnostic work-up of migraine patients with WMHs, being highly specific for MS (Absinta et al., 2012; Lapucci et al., 2019; Sinnecker et al., 2019).

Much attention has been paid to the differential diagnostics between the migraine aura, especially when presenting as negative scotoma, and the acute ischemic stroke. In fact, on admission to the emergency room, 1 to 41% of patients presenting with stroke-like symptoms are events that mimic a stroke but are not caused by an ischemic brain event (Merino et al., 2013). Among the latter, the migraine aura is the third most frequent cause after epileptic aura and psychiatric disorders (Terrin et al., 2018). An accurate ophthalmological evaluation, including best-corrected visual acuity, slit-lamp biomicroscopy, intraocular pressure measurement, and indirect ophthalmoscopy, by excluding optical media, retinal, or optic nerve diseases, can help in the differential diagnosis. It has recently been observed that perfusion CT imaging can help in the decision-making process leading to the differential diagnosis of symptoms mimicking a stroke and, therefore, can direct to appropriate treatment (Nieuwkamp et al., 2010; Hansen et al., 2011; Miller and Goldberg, 2012; Campbell et al., 2013; Shah et al., 2013; Angermaier et al., 2014; Rath et al., 2017; Ridolfi et al., 2018; Granato et al., 2020). Diagnostic accuracy can be further improved by refining the diagnostic criteria of transient ischemic attacks, which can help to separate them from mimics (Lebedeva et al., 2018; Dolmans et al., 2019).

Over the last decades, the use of neuroimaging techniques has improved our understanding of the pathophysiology of migraine and provided new insights into the mechanisms underpinning comorbid conditions (Messina R. et al., 2018). A recent study using transcranial sonography reported that migraine patients with depression had a decreased echogenicity of the raphe nuclei. Significant associations between raphe hypoechogenicity and depression have been described in different neurological diseases, supporting their role in the development of depression (Tao et al., 2019). There is evidence showing that migraine patients have functional and structural alterations of limbic areas with a key role in the regulation of mood and affect and in the processing of the emotional aspects of pain (Maizels et al., 2012). Interestingly, a specific involvement of these regions has been demonstrated in migraine patients with anxiety or depression. Functional alterations of the hippocampus (Liu et al., 2015) and thalamus (Wei et al., 2019) have been associated with the presence of anxiety in patients with migraine. Using diffusion tensor imaging, a technique that allows exploring the microstructure of brain WM tracts *in vivo*, Li and colleagues (Li et al., 2011) have shown alterations of the corpus callosum in migraine patients with anxiety or depressive disorder. Similar alterations were also found in the bilateral corona radiata, superior longitudinal fasciculus, thalamic radiation, and internal and external capsules in migraine patients with depression. All these tracts connect different brain regions involved in emotional processing, and their involvement has been described in studies of patients with psychiatric conditions (Yu et al., 2013).

Recent imaging evidence has shown common functional and structural imaging patterns in migraine and RLS, supporting shared pathophysiological mechanisms between these two conditions. Abnormalities of dopaminergic neurons, such as the substantia nigra, and volumetric alterations of the middle frontal gyrus have been demonstrated in patients with comorbid migraine and RLS (Yang et al., 2018; Aldemir et al., 2020). Dysfunction of sensorimotor, attentive, and limbic brain networks is also common to migraine and RLS (Yang et al., 2018).

It has been suggested that increased neuronal excitability and CSD might play a role in the comorbidity between migraine and epilepsy (Nye and Thadani, 2015). So far, only one imaging study has explored imaging biomarkers that might explain the coexistence of these two conditions. Huang and colleagues showed microstructural alterations in the medial lemniscus and cerebellar peduncles in patients with epilepsy and comorbid migraine, suggesting that trigeminal and cerebellar alterations might explain the occurrence of migraine in patients with epilepsy (Huang et al., 2017). Further studies including a larger sample of patients are needed to better understand the mechanisms mediating comorbidities in migraine.

## Conclusion and Perspectives

It is clear from the amount of studies reviewed here that migraine disorders are comorbid with a plethora of pathologies, not only of the CNS (see Table 1). This relationship is always two-way, with migraine patients most frequently affected by comorbidity, just as migraine is frequently comorbid with the pathology under examination.

**Table 1:** List of the most frequent pathologies showing two-way comorbidity with migraine and their supposed pathophysiological mechanisms of comorbidity.

**Comorbid condition**	**Pathologies**	**Genetic substrate**	**Pro-inflammatory**	**Cortical dysexcitability/CSD**	**Energetic failure**
Cerebrovascular dysfunction	Stroke	X	X	X	X
Metabolic and endocrine comorbidities	Diabetes, obesity, insulin resistance, hypothyroidism, and endometriosis	X	X	X	X
Epilepsy	Benign occipital epilepsy of childhood with occipital paroxysms and benign rolandic epilepsy	X		X	
Psychiatric disorders	Major depressive disorder, bipolar disorder, post-traumatic stress disorder, and anxiety disorder	X	X	X	
Other pain syndromes	Fibromyalgia, chronic low-back pain, pain accompanying dysmenorrhea, and temporomandibular disorder		X	X	
Sleep-related disorders	Insomnia, sleep-disordered breathing, restless legs syndrome, narcolepsy, advanced sleep phase, and parasomnias			X	
Gastrointestinal disorders	Periodontitis, gastroesophageal reflux disease, *Helicobacter pylori* infection, hepatobiliary disorders, celiac disease, irritable bowel syndrome, inflammatory bowel disease, and constipation	X	X		
Immunological disorders	Multiple sclerosis, systemic lupus erythematosus, antiphospholipid syndrome, primary Sjögren's syndrome, rheumatoid arthritis, and atopic diseases	X	X		

*CSD, cortical spreading depression*.

**Figure 1:**
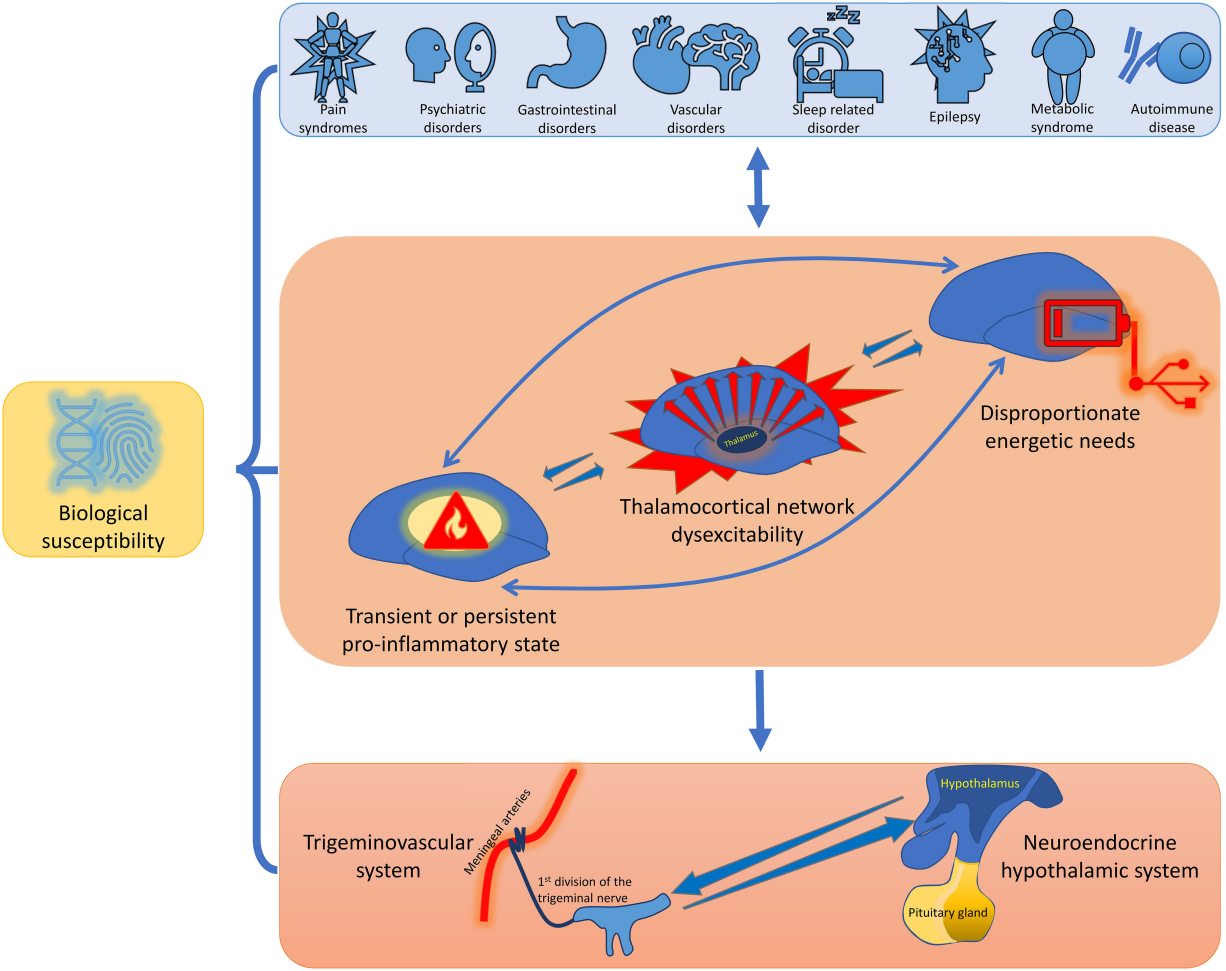
Schematic representation of the pathophysiological model of bidirectional comorbidity between migraine and other medical conditions. A biological susceptibility, constituted by both nuclear and mitochondrial genomic peculiarities, can predispose to different clinical pathological conditions, to the propensity to some physiological mechanisms, and to the lower the activation threshold of some brain structures. Several pathologies can be comorbid with migraine, including neurological, psychiatric, cardio- and cerebrovascular, gastrointestinal, metaboloendocrine, painful, and immunological conditions. The variable combination of thalamocortical network dysexcitability, of multi-organ transient or persistent pro-inflammatory state, and of disproportionate energetic needs induced and promoted by the additive comorbid pathologies may be causative mechanistic factors of the activation of an ample defensive system that includes the trigeminovascular system in conjunction with the neuroendocrine hypothalamic system.

Overall, we can argue that the bidirectional mechanisms that are likely to underlie this extensive comorbidity between migraine and other medical manifestations are manifold (Figure 1). Genetic non-modifiable factors are likely to be a protagonist, with multiple genes playing a role in different areas such as neurotransmission, synaptic plasticity, pain regulation, vascular function, and energetic metabolism. On this genetic basis, modifiable additive factors, such as those that may disturb the normal cerebral homeostatic equilibrium (emotional dysregulation, alterations in wakeful sleep rhythm, incorrect dietary regimes that may increase body weight, hormonal imbalances, musculoskeletal alterations, abnormal work rhythms, and substance abuse) can also play an important role in both setting the cyclical migraine threshold and favoring other medical conditions.

The variable combination of thalamocortical network dysexcitability, of multi-organ transient or persistent pro-inflammatory state, and disproportionate energetic needs induced and promoted by the additive comorbid pathologies, may be causative mechanistic factors of the activation of an ample and diffuse defensive system that includes the trigeminovascular system in conjunction with the neuroendocrine hypothalamic system. The latter, through vagal and spinal extrinsic primary afferent neurons, is involved in coordinating appropriate behavioral responses to aversive and threatening stimuli (Grafe et al., 2017; James et al., 2017). The final product of the activation of this defensive system is the triggering of migraine attack, which sets the alarm on. Therefore, put into a cybernetic system like that of the human organism, migraine pain can be considered the vent valve that keeps the system in stable equilibrium and prevents excessive depletion of energy reserves. On this line of thinking, this could be considered an evolutionary strategy of our brain to try to re-establish a condition of normality and entice or force the patient in search of rest, avoidance of sensory overstimulation, and abstention from food, drink, and other potentially threatening and emotionally distressing behaviors that could continue to compromise the subject in his/her entirety. This strategy of the brain is designed to maintain its homeostasis by regulating homeostatic needs, such as normal subcortico-cortical excitability, energy balance, osmoregulation, and emotional response (Coppola et al., 2021).

Some studies pointed out the headache clinical features and response to acute and preventive treatment can have only minor differences in the two sexes (Vetvik and MacGregor, 2017), but others reported a clear sex disparity in migraine comorbidity (Tietjen et al., 2007; Jensen and Stovner, 2008; Le et al., 2011). Overall, the prevalence of comorbid conditions seems to reflect their epidemiology, with women more frequently affected by psychiatric and immune-mediated disorders and men by vascular and other somatic diseases. Similarly, migraine patients present more often psychological distress at younger ages while somatic comorbidities later in life. However, altogether, migraineurs are affected more frequently by other conditions than age-matched controls, suggesting anticipation of the onset in the disease history (Buse et al., 2020).

Overall, all this implies that the treatment of migraine should always involve a multidisciplinary approach, aimed at identifying and, if necessary, eliminating possible risk and comorbidity factors. This necessarily means that action should be taken as early as possible in life, both as children and as adults when migraine is still episodic. This is to avoid the evolution toward first a chronic form and then toward pharmacological resistance. This educational-behavioral process not only could favor the response to drugs for the attack and prophylaxis but could also allow the therapy to be better tailored to the individual patient.

## Data Availability Statement

The raw data supporting the conclusions of this article will be made available by the authors, without undue reservation.

## Author Contributions

CA and GC contributed to the discussion of content, review, and/or editing of the manuscript before submission. All authors researched data for and participated in the writing of the article and proofread the final manuscript before submission.

## Conflict of Interest

The authors declare that the research was conducted in the absence of any commercial or financial relationships that could be construed as a potential conflict of interest.
